# Hepatic Dystychoma: A Five Year Experience

**DOI:** 10.1155/1991/96304

**Published:** 1991

**Authors:** J. M. Little, Arthur Richardson, Noel Tait

**Affiliations:** Department of Surgery Westmead Hospital Westmead NSW 2145 Australia

## Abstract

In 5 years, 64 solid hepatic lesions have been referred for diagnosis and management which have been found unexpectedly on organ imaging in well patients. We have called this lesion a “dystychoma”.

Patients have undergone a two phase investigation programme which allows a diagnosis without admission to hospital in about 50% of cases. About three quarters of patients (47/64) have had nonneoplastic lesions, and about half (33/64) have had haemangiomas. About one patient in four (17/64) has had a neoplasm, and the neoplasm has been malignant in about one in six (11/64) of all patients.

We stress the need to pursue the diagnosis in these patients. There were no reliable clinical, biochemical or imaging characteristics which individually distinguished benign from malignant lesions. Age over 55 years, an enlarged liver or a palpable liver mass and a raised serum alkaline phosphatase were all significantly more frequent with malignant tumours. The risk of malignancy rose with the number of risk factors, and all patients with all three risk factors had malignant tumours.

Only 11 of the 64 patients were judged to have benefited by significant increase in quality or quantity of life as a result of what was frequently inappropriate organ imaging. There is no strong argument for replacing history taking and physical examination by CT scanning, ultrasound examination or other organ imaging.


*HPB Surgery*, 1991, Vol. 4, pp 291–297
Reprints available directly from the publisher
Photocopying permitted by license only

© 1991 Harwood Academic Publishers GmbH
Printed in the United Kingdom

### HEPATIC DYSTYCHOMA: A FIVE YEAR EXPERIENCE

J.M. LITTLE, ARTHUR RICHARDSON and NOEL TAIT
*Department of Surgery, Westmead Hospital, Westmead NSW 2145, Australia*

*(Received 12 April 1991)*

In 5 years, 64 solid hepatic lesions have been referred for diagnosis and management which have been found unexpectedly on organ imaging in well patients. We have called this lesion a "dystychoma".

Patients have undergone a two phase investigation programme which allows a diagnosis without admission to hospital in about 50% of cases. About three quarters of patients (47/64) have had non-neoplastic lesions, and about half (33/64) have had haemangiomas. About one patient in four (17/64) has had a neoplasm, and the neoplasm has been malignant in about one in six (11/64) of all patients.

We stress the need to pursue the diagnosis in these patients. There were no reliable clinical, biochemical or imaging characteristics which individually distinguished benign from malignant lesions. Age over 55 years, an enlarged liver or a palpable liver mass and a raised serum alkaline phosphatase were all significantly more frequent with malignant tumours. The risk of malignancy rose with the number of risk factors, and all patients with all three risk factors had malignant tumours.

Only 11 of the 64 patients were judged to have benefited by significant increase in quality or quantity of life as a result of what was frequently inappropriate organ imaging. There is no strong argument for replacing history taking and physical examination by CT scanning, ultrasound examination or other organ imaging.

KEY WORDS: Liver tumours, diagnosis, epidemiology

### INTRODUCTION

In 1990, members of this Unit drew attention to a new clinical entity — a solid lesion found unexpectedly by organ imaging in the liver of a relatively well patient[1]. We originally named this a hepatic "incidentaloma", but have felt that the finding has not been truly incidental in some patients who have had symptoms compatible with liver pathology. We have been advised by a skilled medical etymologist that the term "dystychoma" — meaning "unlucky tumour" — might be more appropriate.

We here report on an extended experience with this entity over 5 years, and make further suggestions about the epidemiology, diagnosis and management of the lesion.

### MATERIALS AND METHODS

A dystychoma is defined as an unexpected solid lesion in the liver of a patient with a Karnofsky index of 80% or more, detected or confirmed by organ imaging using computerised tomography (CT) or ultrasound[1]. We have not included cystic lesions, nor solid lesions detected in patients included in screening or follow-up programmes for chronic hepatitis, bowel or breast cancer or melanoma.

We have recorded information on oral contraceptive use, symptoms, physical signs, the size and number of lesions in each patient. Patients have undergone a standard two-phase investigation programme, previously described[1]. In the Out Patient Department, we have obtained liver function tests, hepatitis serology, alpha foetoprotein (AFP) and carcinoembryonic antigen (CEA) levels and a labelled red cell blood pool scan. The red cell scan has been confirmed as a reliable, sensitive and specific diagnostic test for hepatic haemangioma[2].

If no diagnosis has been made when these results were available, patients have been admitted for 2 days to hospital for hepatic angiography and fine needle aspiration cytology (FNA). If it has seemed appropriate, we have sought a primary tumour when the FNA has suggested that the hepatic lesion is a secondary tumour.

### RESULTS

### *General*

In the 60 month period ending December 1990, we saw 64 patients fulfilling the defining criteria. Their median age was 47 years, with a range of 17 to 81.

Forty five of the 64 were women, a significance preponderance (chi-square 10.56, $p < .005$). Seventeen of the 45 women had taken the oral contraceptive for more than a year.

### *Symptom Classification*

Symptoms were graded to 3 levels. Level 0 represented no symptoms referable to the abdomen. Level 1 represented non-specific abdominal discomfort or pain, without hepatobiliary localisation. Level 2 represented symptoms consistent with hepatobiliary disease. Symptoms were approximately equally distributed between the 3 levels (Table 1).

**Table 1** Symptom classification

| *SYMPTOM SCORE* | *NUMBER* |
|---|---|
| 0 - NO RELEVANT SYMPTOMS | 20 |
| 1 - NON-SPECIFIC ABDOMINAL | 22 |
| 2 - HEPATOBILIARY | 22 |

### *Physical Signs*

Physical signs were also graded 0 to 2, 0 — representing no findings of hepatic pathology, 1 — a palpable liver edge without an apparent liver mass and 2 — a palpable liver mass. The majority of patients had no relevant physical signs (Table 2).

**Table 2** Physical signs

| *SIGN SCORE* | *NUMBER* |
|---|---|
| 0 - NO HEPATIC SIGNS | 51 |
| 1 - PALPABLE LIVER | 7 |
| 2 - PALPABLE MASS | 6 |

### *Liver Function Tests*

In 3 instances, complete liver function tests were not available. Thirty eight of the remaining 61 patients had normal liver function tests (serum bilirubin, serum alkaline phosphatase [SAP], serum alanine-leucine transaminase [ALT], serum gamma-glutamyl transpeptidase [gamma GT] and serum albumin). One or more tests were abnormal in 23 patients.

The SAP was less frequently elevated, raised levels being detected in only 13 of the 61 patients for whom full liver function tests were available.

### *Tumour Markers*

The CEA was raised in 8 of 52 patients in whom its level was estimated. One of these raised levels appears to represent a false positive, since the patient had a haemangioma with no evidence of primary or secondary colorectal carcinoma during a 2 year follow-up. The other 7 reflected the presence of subsequently confirmed colorectal secondary carcinoma in the liver.

AFP was estimated in 55 patients and was elevated in 4. 2 had HCC confirmed, but in the other 2 these elevations appeared to be false positives. Of the remaining 51 patients with normal levels, 2 were found to have hepatocellular carcinoma (HCC).

### *Hepatitis Serology*

Hepatitis B serology was obtained for 56 patients, and was positive in 3. Hepatitis C serology became available only recently, and there are too few results to report.

### *Number and Size of Lesions*

Lesions were single in 46 patients, multiple in 18. The size of individual lesions varied from 1 to 13 cm in greatest diameter as measured on CT or ultrasound. The median greatest diameter was 5 cm.

### *Blood Pool Scans*

Blood pool scans were obtained for 58 patients, and were diagnostic of haemangioma in 28. Five additional haemangiomas were diagnosed by arteriography.

### *Diagnoses*

There were 47 non-neoplastic lesions — 33 haemangiomas, 7 focal nodular hyperplasias (FNH), 5 focal fatty infiltrations and 1 each tuberculous abscess and penetrating peptic ulcer. There were 6 benign neoplasms — 5 adenomas and 1 leiomyoma. The remaining 11 patients had malignant neoplasms — 7 colorectal metastases and 4 hepatocellular carcinomas (HCC). Thus, 47 (74%) had non-neoplastic lesions, while 11 (17%) had malignancies.

These figures are summarised in the flow chart of Figure 1.

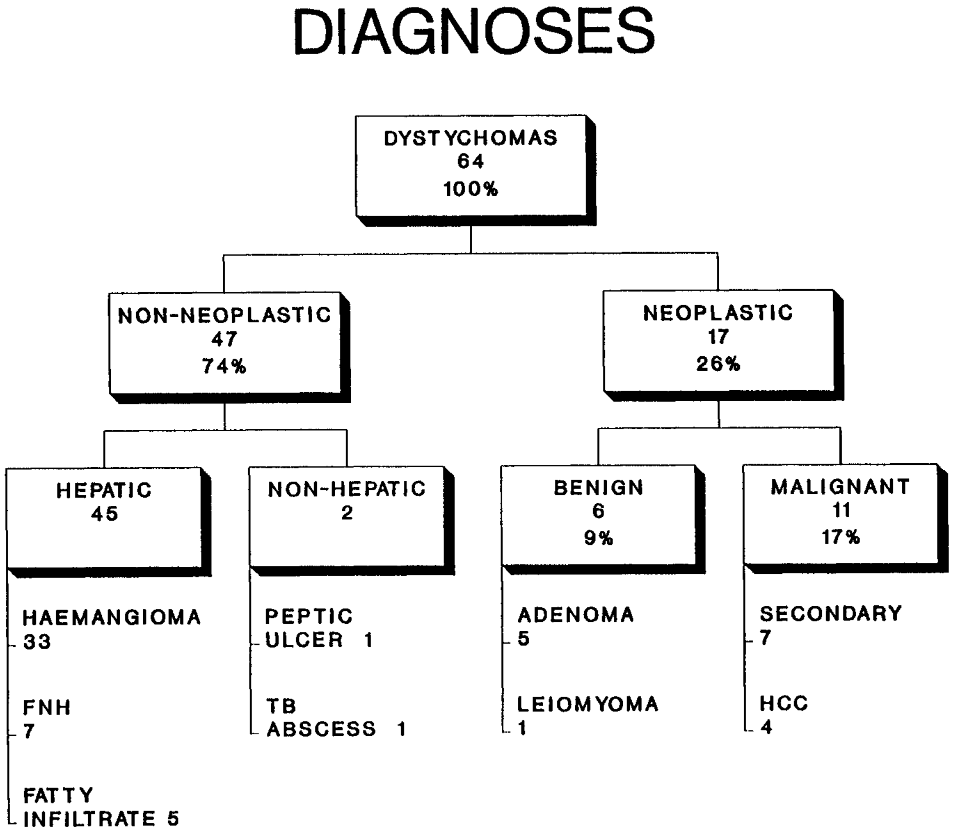

**Figure 1** Flow chart outlining the distribution of diagnoses among the liver lesions.

### *Diagnostic Phase*

A diagnosis was made during the Out Patient phase in 31 patients, the remaining 33 requiring admission to hospital.

### *Predictors of a Benign or Malignant Tumour*

Univariate analysis demonstrated that patients with malignant tumours were significantly older than those with benign lesions (62 years compared with 43 years, $p < .0001$, Mann Whitney). Eight of 13 with a palpable liver or palpable liver mass had malignancies, compared with 5 of 51 without physical signs of liver enlargement ($p < .0001$, Fisher test). Eight of 13 with an elevated SAP had malignancies, compared with 5 of 48 with measured normal levels ($p < .0001$, Fisher test). Sex, the presence or absence of symptoms, the diagnosis of hepatitis, singularity or multiplicity of lesions and their size did not differ between the groups with benign and malignant diagnoses.

The influence of small size was examined in more detail. None of 8 patients with lesions smaller than 3 cm maximum diameter had malignant tumours, compared with 1 of 13 with lesions from 3–3.9 cm. Thus, only 1 of 21 lesions less than 4 cm in maximum diameter was malignant, compared with 10 of 43 lesions measuring 4 or more cm in maximum diameter. None of these differences reached statistical significance.

When the sizes of lesions which were not haemangiomas were compared, 1 of 7 lesions less than 4 cm maximum diameter proved to be malignant, compared with 10 of 24 larger lesions. This difference was not statistically significant ($p = .3717$, Fisher test).

The three factors that were found to distinguish statistically between benign and malignant lesions were arbitrarily given equal weight and combined in a single score. Age less than 55 scored 0, age over 55 scored 1; impalpable liver scored 0, palpable liver or liver mass 1; and normal SAP scored 0, raised SAP 1. The total score was obtained by simple addition for each patient. There were no malignancies among patients with total scores of 0; there were 2 among the 18 patients scoring 1; 3 in the 4 patients scoring 2; and all 6 who scored 3 had malignant tumours.

### *Patient Benefit*

An attempt has been made to judge whether each patient has benefited by improvement in quality or quantity of life, by assessing symptoms before and after management of the lesion which prompted referral and by assessing the likely outcome without intervention. Eleven of the 64 patients have unequivocally benefited, from relief of pain in 7 and from the removal of life threatening pathology in 4. We have, of course, managed to give reassurance to the majority of patients whose anxiety was created by the organ imaging investigation.

### DISCUSSION

The clinical problem presented by the chance finding of a solid lump in the liver on organ imaging was described by this unit in 1990 under the name "hepatic incidentaloma"[1], but we recognise that the term "incidentaloma" is misleading. The term "dystychoma" — meaning "unlucky tumour" — was suggested to us by Dr David S. Johnson, and seems more appropriate to the conditions under which these lesions are found. Once again, we have been impressed with the frequency with which organ imaging investigations are being ordered in our medical community for conditions that seem quite inappropriate. Twenty of our 64 patients had no symptoms that could be associated with the liver or biliary system. They had all presented with self-limiting illnesses, unrelated to their liver lesions. The great majority (51 of 64) had no physical findings to suggest liver pathology.

The two phase investigation protocol previously described[1] continues to work reasonably well, and about half our patients have been given a diagnosis and reassured without admission to hospital because the red cell scan was positive for haemangioma. The remainder have needed admission for angiography and, usually, FNA. Some have needed laparotomy and open biopsy, because the FNA has produced dysplastic liver cells which did not distinguish between focal nodular hyperplasia (FNH), adenoma and HCC.

It has been suggested that HIDA scanning might be added to this protocol of investigation, in order to increase the level of confidence in distinguishing benign from malignant lesions. Calvet and colleagues[3], however, have demonstrated that some well differentiated HCC's take up HIDA. Biersack *et al.*[4] have noted uptake by FNH, and Strashun and Goldsmith[5] by metastatic breast carcinoma. It seems unlikely that HIDA scanning will do anything to clarify the diagnostic confusions.

The majority of lesions (74%) were not neoplastic, and the commonest single diagnosis was haemangioma (52%). Unfortunately, 26% of lesions were neoplastic, and 17% of the total were malignant. The benign tumours must at least be followed carefully at 6 month intervals to ensure that they are not increasing in size[6,7]. Severely symptomatic benign lesions should be removed if it is safe to do so.

This study has confirmed our earlier advice[1] that the clinician should seek the highest order diagnosis possible, since there are no clinical or biochemical features that reliably distinguish benign from malignant lesions. There are some clinical guides which help, but none of them seem wholly reliable. Older patients — more than 55 years — were more likely to have malignancy. Those with enlarged livers or palpable masses in the liver were more likely to have malignancy, but 5 of 13 with palpable livers or liver masses had benign lesions. A raised SAP was also an indicator of malignancy, but once again 5 of 13 with raised SAP had benign lesions. The combination of these risk factors indicated an increased chance of malignancy. The absence of all factors was associated with no incidence of malignancy; the presence of 1 factor carried 1 chance in 9 of malignancy; two factors, 3 chances in 4; while all 3 factors were associated with 6 malignancies in 6 patients. Small size (less than 4 cm) was associated with a low incidence of malignancy (1 in 21 patients), but the difference from the incidence of 10 in 43 with larger lesions was not statistically significant.

There is no doubt that clinicians will see more of this and similar problems, created by the ready access to powerful organ imaging devices. One can debate the validity of performing a CT scan for an episode of self-limited abdominal pain, but the test will be done unless governments or the profession introduce limits or guidelines. The finding that only 11 (17%) of the 64 patients in this present study definitely benefited from the discovery of their liver lesion does not support the present unselective use of organ imaging to replace history taking and physical examination.

### *References*

1. Little, J.M., Kenny, J. and Hollands, M.J. (1990) Hepatic incidentalomas: a modern problem. *World J. Surg.*, **14**, 448–451
2. Farlow, D.C., Chapman, P.R., Gruenwald, S.M., Antico, V.F., Farrell, G.C. and Little, J.M. (1990) Investigation of focal hepatic lesions: is tomographic red blood cell imaging useful? *World J. Surg.*, **14**, 472–477
3. Calvet, X., Pons, F., Bruix, J., Bru, C., Lomena, F., Herranz, R., Brugera, M., Faus, R. and Rodes, J. (1988) Technetium-99m DISIDA hepatobiliary agent in diagnosis of hepatocellular carcinoma: relationship between detectability and tumour differentiation. *J. Nucl. Med.*, **29**, 1916–20

4. Biersack, H.J., Thelen, M., Torres, J.F., Lackner, K. and Winkler, C.G. (1980) Focal nodular hyperplasia of the liver as established by 99m-Tc-sulfur colloid and HIDA scintigraphy. *Radiology*, **137**, 187–190
5. Strashun, A. and Goldsmith, S. (1981) Increased focal uptake of Tc-99m-IDA hepatobiliary agent by a liver metastasis. *Clin. Nucl. Med.*, **6**, 295–296
6. Foster, J.H. (1977) Primary benign solid tumours of the liver. *Am. J. Surg.*, **133**, 536–541
7. Foster, J.H. (1982) Benign liver tumours. *World J. Surg.*, **6**, 25–31

(*Accepted by S. Bengmark 12 April 1991*)

